# News and Miscellany

**Published:** 1905-04

**Authors:** 


### News and Miscellany.

Dr. L. L. Loggins, of Lufkin, was shot and killed in a fight recently at that place.

---

The president of the faculty of the New York Polyclinic Medical School and Hospital, on Tuesday, December 20th, gave a reception to the members of the teaching staff, the board of trustees, and many of their friends, to celebraie the event of the liquidation of a second mortgage of about $40,000, which was accomplished by the personal donations of the members of the staff. This action was applauded by a member of the board of trustees in a material way by a personal donation of $20,000. It is hoped that this generous contribution will be productive of other donations, and that the new building fund will soon be of substantial size.

---

SEMI-CENTENNIAL OF FREDERICK STEARNS & CO.—This great house of manufacturing pharmacists, founded in 1854 by Mr. Frederick Stearns, celebrated its fiftieth anniversary recently, and issued a special number of "The New Idea" in commemoration thereof. It gives a historical sketch of the house—the pioneer of the West—and much valuable and interesting matter in connection with the evolution of the business from small beginnings to the immense proportions of today. The founding and development of the antitoxin serum industry is particularly interesting. It is illustrated with half-tones of the founder and members of the firm. Mr. J. W. T. Knox, of the firm, will send you a copy of this memorial edition on request.

---

THE popular, gifted and original Dr. Emery Lanphear, so long the brains and energy behind the *American Journal of Surgery and Gynecology,* has retired from the field of journalism, to give his entire attention to his general and special practice. I consider his retirement a distinct loss to the guild. We will miss his pungent paragraphs, his keen witticisms, and his caustic criticism of things that do not go to suit him. We will miss his peculiar spelling,— he will be "misst" in many ways. *Vale.* Good luck to you, *mon chere ami.*

---

DR. J. MACDONALD, JR., writes: I have purchased all rights in the *American Journal of Surgery and Gynecology,* and with the April number this journal, thoroughly modernized and largely increased in circulation, will be issued from New York as the *American Journal of Surgery.* In this undertaking I will have the contributory co-operation and support of such well-known surgeons and teachers as: Robt. T. Morris, Professor of Surgery, New York Post-Graduate School; Howard Lilienthal, Visiting Surgeon, Mt. Sinai Hospital, New York; J. P. Tuttle, Professor Rectal

Diseases, New York Polyclinic; Jas. T. McKernan, Professor Nose and Throat, New York Post-Graduate School; Saml. G. Gant, Professor Rectal Diseases, New York Post-Graduate School; Augustin H. Goelet, Professor Gynecology, New York Clinical School of Medicine; C. Wendall Phillips, Professor of Diseases of the Ear, New York Post-Graduate School; Ferdinand C. Valentine, New York, who, with others, will assist me in making a practical surgical journal, which, in point of interest and usefulness, will represent all that years of experience backed by ample capital can produce.

---

THE BOARD OF MEDICAL EXAMINERS for the State of Texas (Regular) will hold its next meeting in Austin, Texas, May 2, 3, 4, and 5, 1905, for the examination of applicants and transaction of other business. For further information, address the secretary.

J. T. WILSON, President,

Sherman, Texas.

M. M. SMITH, Secretary,

Austin, Texas.

---

ANOTHER MEDICAL COLLEGE FOR DALLAS.—Dr. Arch B. Keatley announces that he will start "The Eclectic Medical and Surgical University of Dallas," April 10th, inst., and that he has selected "a staff of medical professors which will be paid $100 per month each for their superior services," and that "in a very short time" it "will be the largest and most worthy medical institution in the South." (Neither Tulane, nor the Texas Medical, nor Vanderbilt will be in a little bit.) The doctor says, "I have charge of the finance and management." "The money is in store to make the school a great and noble institution." "Will run eight weeks as post-graduate at a cost of $50 each per student." "Send me $5 matriculation, leaving you $45 to pay on entering the school."

---

NO ONE thinks of going to a lawyer or engineer or architect without his fee; yet doctors are supposed to be under a moral obligation to give their services freely to the ill and injured. This they do, to their honor, relieving pain and suffering by the expenditure of their time and skill, often with no hope or possibility of payment. Yet their protests against perils to the general health are frequently resented, and treated as the selfishness of a profession aiming to thrust itself in everywhere. This is an attitude of mind really as bewildering as outright superstition. It is puzzling to find distrust provoked by what is really the crowning proof of disinterestedness; since, as it has been said, the devotion of the medical profession to the cause of public health is really a way of making its own extinction its final goal.—*New York Evening Post.*

---

MISSED NOTHING BUT HIS WATCH.—An old darkey was taken ill, says the *New York Times,* and called in a physician of his own race. After a time, as there were no signs of improvement, he asked for a white doctor. Soon after arriving, Dr. ——— felt the old man's pulse, and then examined his tongue. "Did your other doctor take your temperature?" he asked. "I don't know, boss," replied the sick negro, "I hain't missed anything but my watch as yit."

---

$1500 CASH buys fine home in famous onion district of Southwest Texas. In county seat; 1200 inhabitants; $2000 practice. Opposition, one other doctor. Address, Dr. D., care TEXAS MEDICAL JOURNAL.

---

FOR SALE.—Quick for cash, one Peerless cash register, good order, $60 machine, $35.

One $18 Betz hot-air apparatus for knee, leg, arm or body, $10.

One $24 combined galvanic and faradic battery, McIntosh, in good order, $12.50.

Correspondence solicited and machines will be shipped for trial.

LYLE G. THORNTON, M. D.,
West Point, Texas.

